# Propiedades psicométricas de la Escala Observacional de la Marcha del Sujeto con Amputación de la Extremidad Inferior

**DOI:** 10.23938/ASSN.1152

**Published:** 2026-02-10

**Authors:** Aránzazu Cortés Rodríguez, David Hernández Herrero, Isabel Mª Alguacil Diego, Ángela Aguilera Rubio, Mónica González Nuño, Francisco Molina Rueda

**Affiliations:** 1 Centro de Fisioterapia Fisiortam Madrid España; 2 Hospital Universitario La Paz Madrid España; 3 Departamento de Fisioterapia, Terapia Ocupacional, Rehabilitación y Medicina Física Facultad de Ciencias de la Salud Universidad Rey Juan Carlos Alcorcón Madrid España; 4 Laboratorio de Análisis del Movimiento, Biomecánica, Ergonomía y Control Motor (LAMBECOM) Facultad de Ciencias de la Salud Universidad Rey Juan Carlos Alcorcón Madrid España

**Keywords:** Amputación de miembro inferior, Cambio Mínimo Detectable, Escala Observacional de la marcha, Fiabilidad, Validez de constructo, Lower Limb Amputation, Minimal Detectable Change, Observational Gait Scale, Reliability, Construct Validity

## Abstract

**Fundamento:**

El objetivo es analizar las propiedades psicométricas de la Escala Observacional de la Marcha del Sujeto con Amputación de la Extremidad Inferior (OMSAEI): fiabilidad intra e interobservador, cambio mínimo detectable (CMD_95_) y validez de constructo.

**Metodología:**

Se realizó un estudio observacional en personas con amputación del miembro inferior (AMI) unilateral. Las grabaciones de la marcha se realizaron en condiciones controladas y fueron evaluadas por dos profesionales que aplicaron la escala OMSAEI organizada en dos secciones: 1- evaluación de patrones cinemáticos, centrados en el análisis de la movilidad articular, y 2- evaluación de parámetros espaciotemporales. Se analizaron el coeficiente de correlación intraclase (CCI), los gráficos de Bland-Altman y la correlación de Pearson con la escala de Houghton.

**Resultados:**

Participaron 37 personas, 56,8%, mujeres y edad media 45,6 años (DE=13,8). La fiabilidad intraobservador fue excelente (CCI total=0,996; sección 1=0,988; sección 2=0,995), con un CMD_95_ de 0,12 puntos. La fiabilidad interobservador también fue excelente (CCI total=0,987; sección 1=0,966; sección 2=0,986), con un CMD_95_ de 0,36 puntos. Los gráficos de Bland-Altman mostraron límites de acuerdo estrechos: ±1,4 puntos (intra) y ±2,28 puntos (inter). La validez de constructo fue alta y significativa (r= -0,773; p<0,001), indicando una fuerte correlación inversa con la escala de Houghton.

**Conclusiones:**

La escala OMSAEI es una herramienta fiable y válida para realizar una evaluación objetiva y estructurada del patrón de marcha en personas con AMI unilateral. Su diseño específico y sus excelentes propiedades psicométricas la convierten en un instrumento útil tanto en contextos clínicos como de investigación.

## Abstract (en)

**Background:**

The aim of this study was to examine the psychometric properties of the Observational Gait Scale for Persons with Lower Limb Amputation, including intra- and inter-rater reliability, minimal detectable change, and construct validity.

**Methods:**

An observational study was conducted in individuals with unilateral lower limb amputation. Gait was recorded under standardized conditions and independently assessed by two trained evaluators using the Observational Gait Scale for Persons with Lower Limb Amputation, which comprised two sections: 1 - assessment of kinematic gait patterns focused on joint mobi-lity, and 2 - assessment of spatiotemporal parameters. Intraclass correlation coefficient, Bland-Altman plots and Pearson’s correlation with the Houghton Scale were used.

**Results:**

Thirty-seven participants were included, 56.8% women and mean age 45.6 years (SD=13.8). Intra-rater reliability was excellent for the total score (ICC = 0.996), section 1 (ICC = 0.988), and section 2 (ICC = 0.995) with an MDC_95_ of 0.12 points. Inter-rater reliability was also excellent (total ICC = 0.987; section 1 = 0.966; section 2 = 0.986), with an MDC_95_ of 0.36 points. Bland-Altman analysis showed narrow limits of agreement (±1.4 points intra-rater and ±2.28 points inter-rater). Construct validity was strong and statistically significant (r= -0.773; p <0.001).

**Conclusions:**

The Observational Gait Scale for Persons with Lower Limb Amputation is a reliable and valid tool for assessing an objective and structured evaluation of gait patterns in individuals with unilateral lower limb amputation. Its design, excellent reliability and strong construct validity support its use in both clinical practice and research.

## INTRODUCCIÓN 

La evaluación objetiva de la marcha en personas con amputación de miembro inferior (AMI) resulta fundamental en el ámbito clínico, dada su repercusión en la autonomía funcional y en la calidad de vida del paciente. La amputación compromete el control motor, especialmente la estabilidad en bipedestación y el patrón de marcha, lo que limita el desempeño de las actividades básicas de la vida diaria, afectando negativamente la participación social del individuo^1^^,^^2^, ya mermada en la persona con AMI^3^.

Uno de los objetivos de la rehabilitación es optimizar el uso del componente protésico para restablecer una marcha funcional y una postura estable, minimizando la dependencia de productos de apoyo^4^. No obstante, las alteraciones sensoriomotoras derivadas de la AMI suelen dar lugar a patrones compensatorios que incrementan el coste energético de la marcha, favorecen la fatiga y predisponen a complicaciones como heridas en el muñón o artropatías degenerativas en las articulaciones de los segmentos intactos^5^^-^^7^. La persistencia de estos patrones implica además una sobrecarga del miembro inferior intacto, de las articulaciones conservadas del lado protetizado y de la musculatura del tronco, que debe asumir funciones adicionales de estabilización y propulsión durante la marcha. Entre las manifestaciones clínicas más frecuentes destaca el dolor lumbar crónico relacionado con esta sobrecarga y con las alteraciones biomecánicas asociadas^8^^,^^9^. 

Al permitir objetivar las desviaciones cinemáticas y espaciotemporales, las escalas observacionales de la marcha se consolidan como herramientas esenciales para cuantificar alteraciones en el patrón de marcha^10^, monitorizar la adaptación progresiva del paciente al uso de la prótesis y valorar su evolución clínica durante el tratamiento. 

Por tanto, resulta indispensable disponer de un instrumento fiable y válido de evaluación de la marcha específico para la persona con AMI. Su implementación permitiría homogeneizar los protocolos de investigación, facilitar la comparación de resultados entre estudios y fortalecer la evidencia científica sobre la rehabilitación en personas con AMI.

Molina-Rueda y col^7^ elaboraron una escala específica para la evaluación de la marcha en la persona con AMI a partir del consenso de un comité de expertos que definió el contenido de la escala de acuerdo con las alteraciones de la marcha más frecuentes en la población con AMI y evaluó la validez de contenido de la herramienta, con resultados excelentes. La definición de los ítems, el sistema de puntuación y las instrucciones de la Escala Observacional de la Marcha del Sujeto con Amputación de la Extremidad Inferior (escala OMSAEI) fueron desarrollados a partir del análisis de múltiples instrumentos observacionales previamente validados en el campo del análisis de la marcha como la *Gait Assessment and Intervention Tool* (POGS) o la *Prosthetic Observational Gait Score* (GAIT)^11^^-^^13^, esta última específica para población con AMI pero con propiedades psicométricas no estudiadas en profundidad^7^^,^^13^. 

Consideramos necesario, por ello, realizar un estudio de las propiedades psicométricas de la escala OMSAEI ya que, a pesar de su adecuado desarrollo teórico y validez de contenido, existe un vacío de evidencia empírica sobre su fiabilidad y validez psicométrica. También sería interesante analizar la correlación entre la escala OMSAEI y la escala de Houghton, una herramienta diseñada para evaluar el uso funcional de la prótesis en personas con AMI^14^^,^^15^. Contar con esta información permitiría disponer en el ámbito clínico de un instrumento fiable y válido específico para evaluar la marcha de las personas con amputación.

El objetivo principal de la investigación fue evaluar la fiabilidad intra e interobservador de la escala OMSAEI, mientras que los objetivos secundarios fueron: 1) calcular los valores de sesgo, los límites de acuerdo y el cambio mínimo detectable (CMD_95_) de la escala, y 2) analizar la correlación entre la escala OMSAEI y la escala de Houghton. 

## MATERIAL Y METODOS

### Diseño del estudio 

Se trata de un estudio de propiedades psicométricas realizado entre enero y abril de 2025 en un centro de fisioterapia especializado en la rehabilitación de pacientes con amputación, Fisiortam, ubicado en la Comunidad de Madrid. La recogida de datos se realizó íntegramente en dicho centro.

### Participantes

Se seleccionaron pacientes con AMI mediante muestreo no probabilístico consecutivo. Los criterios de inclusión fueron: 1) mayor de edad; 2) AMI unilateral (transtibial, transfemoral, desarticulación de rodilla o cadera); 3) tiempo mínimo de uso prótesis ≥1 mes; 4) capacidad para deambular al menos seis metros con o sin productos de apoyo. Se excluyeron personas con alteraciones neurológicas, cardiorrespiratorias o locomotoras adicionales que afectaran la marcha, así como dolor incapacitante, procesos infecciosos agudos o deterioro cognitivo que impidiera seguir instrucciones verbales.

### Estimación del tamaño de la muestra

El tamaño muestral se calculó según estudios previos^16^^,^^17^ a partir del coeficiente de correlación intraclase (CCI) y del número de evaluadores, asumiendo un nivel de significación α = 0,05 y una potencia estadística del 80%. Considerando un CCI mínimamente aceptable (p0) de 0,4 y un CCI esperado (p1) de 0,7 -y siguiendo las tablas de contingencia de Walter y col^16^-, el tamaño muestral necesario fue de 33 sujetos para la fiabilidad intraobservador y 21 para la fiabilidad interobservador. Considerando una tasa de abandono del 10%, el tamaño final fue de 37 personas. 

### Procedimiento

Las personas participantes, tras recibir información completa del estudio, firmaron voluntariamente el consentimiento informado, pudiendo revocar su participación en cualquier momento. El estudio fue aprobado por el Comité de Ética de la Investigación de la Universidad Rey Juan Carlos (Madrid, España) (nº de registro interno: 2102201804018). 

La evaluación se inició cumplimentando la hoja de registro inicial: edad, sexo, causa, nivel y lado de la amputación, tipo de prótesis y encaje, uso de productos de apoyo, tiempo de evolución y de uso de la prótesis y presencia de miembro fantasma doloroso. 

A continuación se administró la escala de Houghton, instrumento diseñado para valorar en la frecuencia de utilización de la prótesis, la confianza de la persona con AMI al usarla, y su capacidad para caminar en distintos entornos. Consta de cuatro ítems, cada uno puntuado de 0 a 3; el rango de puntuación es de 0 a 12 puntos, y puntuaciones más altas indican mayor nivel de uso y funcionalidad protésica. Ha mostrado buenas propiedades psicométricas, con evidencia de fiabilidad y validez en población con AMI^14^^,^^15^.

Seguidamente, se pidió a los pacientes que caminaran a su cadencia habitual por un pasillo recto y liso, con una longitud mínima de seis metros. Se grabó la marcha de cada persona con un móvil Samsung S22+, equipado con un sistema de triple cámara trasera que incluye un sensor principal de 50 megapíxeles con apertura f/1.8 y estabilización óptica de imagen, una cámara ultra gran angular de 12 megapíxeles con apertura f/2.2 y un teleobjetivo de 10 megapíxeles con zoom óptico. El dispositivo permite la grabación en resolución 4K a 30 y 60 fotogramas por segundo, así como en resolución Full HD (1080p), lo que asegura una calidad de imagen suficiente para el análisis detallado del patrón de marcha, en los planos anteroposterior y lateral. Para estabilizar la grabación se utilizó un trípode y, para facilitar la correcta visibilidad de los movimientos articulares, se pidió al paciente que llevara ropa ajustada. Se permitió el uso de productos de apoyo.

Dos clínicos especializados en análisis de la marcha observaron de forma independiente las grabaciones y aplicaron la escala OMSAEI^7^, que se organiza en dos secciones: 1) evaluación de patrones cinemáticos, centrados en el análisis de la movilidad articular de cadera, rodilla, tobillo, pelvis y tronco; 2) evaluación de parámetros espaciotemporales, como duración de apoyo, longitud y anchura del paso, velocidad y trayectoria durante las fases de la marcha. Cada ítem puntúa 0 si el patrón es normal, 1 si la desviación es leve, y 2 si es grave. La puntuación total varía de 0 a 35 puntos, donde 35 refleja una alteración grave del patrón de marcha. Ambos evaluadores recibieron entrenamiento previo para homogeneizar criterios. La administración de la escala solo se realizó para el miembro inferior protetizado.

Los evaluadores estaban cegados a valoraciones previas, y la comparación de sus puntuaciones permitió valorar la fiabilidad interobservador. A las dos semanas, uno de ellos repitió la evaluación de los pacientes con AMI para valorar la fiabilidad intraobservador^18^. 

### Análisis estadístico

Las variables clínicas se expresaron mediante media, desviación estándar (DE) y frecuencias. La fiabilidad se calculó mediante el CCI (modelo de efectos mixtos para medidas individuales, acuerdo absoluto), con intervalos de confianza al 95% (IC95%). Valores del CCI <0,50 implican fiabilidad pobre; de 0,50-0,75, moderada; de 0,75-0,90, buena; y >0,90, excelente^17^.

El método de Bland y Altman se utilizó para calcular el nivel de acuerdo entre las dos observaciones realizadas por el observador 1 (fiabilidad intraobservador) y entre las evaluaciones realizadas por los dos observadores (fiabilidad interobservador), estableciendo los límites de tolerancia y esperando que la mayoría de las diferencias entre las observaciones estuvieran entre la media y dos DE de la media^19^.

Además, se realizó un análisis de correlación entre la puntuación obtenida por el observador 1 en la escala OMSAEI y la puntuación en la escala de Houghton (cuya puntuación es mayor a mejor funcionamiento con la prótesis), utilizando el coeficiente de correlación de Pearson*,* cuyo valor oscila entre -1 y +1. Se interpretaron valores absolutos entre 0,7 y 0,9 como correlación fuerte, entre 0,4 y 0,6 como moderada, y entre 0,1 y 0,3 como débil^20^.

El CMD se define como la menor magnitud de cambio que un instrumento es capaz de detectar como real, es decir, un cambio que supera el error de medida inherente al propio instrumento. Se calculó el CMD al 95% de confianza (CMD_95_) de la escala OMSAEI basado en los datos de las observaciones intra- e interobservador, usando la fórmula: *CMD*_*95*_ = 1,96*√2*SEM donde SEM es el error estándar de la medición^21^, que a su vez se obtiene con la fórmula: *SEM*=DE×1−*r*, siendo DE la desviación estándar de las puntuaciones de la prueba y *r* el coeficiente de fiabilidad de la medición, en este caso el CCI. Para el análisis se utilizó el software SPSS. 28.0. 

## RESULTADOS

Participaron 37 personas con AMI unilateral, con ligero predominio femenino y 45,6 años de edad media (rango: 21-71 años). Todas finalizaron el estudio (no hubo pérdidas) y ninguna empleaba productos de apoyo. 

La causa predominante de amputación fue de origen traumático (72,2%), afectando más frecuentemente al lado izquierdo (56,8%) y a nivel transfemoral (48,6%). El 64,8% de participantes presentaba dolor de miembro fantasma. Todos utilizaban pies protésicos con acumulador de energía; los componentes más utilizados fueron las rodillas electrónicas (48,6%), los sistemas de suspensión mediante membrana con válvula (59,4%) y los sistemas de anclaje basados en liner con membrana (64,8%) (Tabla 1).

**Tabla 1:** Características de las personas estudiadas y de sus componentes protésicos por categorías

Características de pacientes (n= 37)	
Edad, media (DE)	45,6 (13,82)
Sexo, n(%)	
Hombres	16 (43,2%)
Mujeres	21 (56,8%)
Tiempo de evolución (meses), media (rango)	46,8 (1-396)
Lado afectado, n(%)	
Izquierdo	21 (56,8%)
Derecho	16 (43,2%)
Causas de amputación, n(%)	
Traumáticas	27 (72,2%)
Tumorales	6 (16,2%)
Vasculares	3 (8,1%)
Infecciosas	1 (5,4%)
Uso diario prótesis (horas), media (rango)	12,7 (6-17)
Nivel de amputación, n(%)	
Transfemoral	18 (48,6%)
Transtibial	13 (35,1%)
Desarticulación de la rodilla	4 (10,08%)
Desarticulación de la cadera	2 (5,4%)
Dolor miembro fantasma, n(%)	24 (64,8%)
Componentes protésicos, n (%)	
Rodilla	24 (64,9)
Electrónica	20 (48,6)
Monocéntrica hidráulica con rotación libre	2 (5,4)
Policéntrica hidráulica	1 (2,7)
Policéntrica	1 (2,7)
Pie	37 (100)
Con acumulador de energía	37 (100)
Cadera	2 (5,4)
Tridimensional hidráulica	1 (2,7)
Monocéntrica con ayuda a la extensión	1 (2,7)
Encaje protésico	
TSB (*Total Surface Bearing*)	14 (37,8)
De contención isquiática	12 (32,4)
Híbrido CI/cuadrangular	4 (10,8)
Para desarticulación de rodilla	4 (10,8)
Cesta pélvica	2 (5,4)
Sin encaje (oseointegración)	1 (2,7)
Sistema de suspensión	
Membrana con válvula	22 (59,4)
Apoyo en crestas iliacas	2 (5,4)
Rodillera con válvula	3 (8,1)
Pin y lanzadera	2 (5,4)
Válvula	3 (8,1)
Receptor de tornillo de torsión	1 (2,7)
Pin, lanzadera y rodillera	1 (2,7)
Membrana, válvula y cierre imantado	1 (2,7)
Membrana, pin, lanzadera y válvula	2 (5,4)
Sistema de anclaje	
*Liner* con membrana	24 (64,8)
*Liner* sin membrana	3 (8,1)
Cinchado con correas	2 (5,4)
Otras combinaciones híbridas	3 (8,1)
*Liner* para pin	3 (8,1)
Tornillo de sujeción	1 (2,7)
Vacío sobre el paquete muscular	1 (2,7)

DE: desviación estándar.

### Análisis de la Fiabilidad y Cambio Mínimo Detectable

El observador 1 obtuvo una puntuación media en la escala OMSAEI de 6,00 puntos (DE=6,16) en la primera sesión y de 6,30 puntos (DE=6,18) en la segunda sesión; el observador 2 obtuvo 6,92 puntos (DE=6,37).

El CCI para la fiabilidad intraobservador fue excelente para la puntuación total y para cada sección de la escala. El CCI para la fiabilidad interobservador fue excelente para la puntuación total y para cada sección de la escala. Aunque los IC95% fueron estrechos para la puntuación total y para para la sección 2, fueron algo más anchos para la sección 1 (Tabla 2).

**Tabla 2:** Fiabilidad intraobservador e interobservador

	Fiabilidad CCI (IC 95%)	
Sección	Intraobservador	Interobservador
1	0,988 (0,977-0,994)	0,966 (0,809-0,988)
2	0,995 (0,990-0,997)	0,966 (0,973-0,993)
Total	0,996 (0,992-0,998)	0,987 (0,933-0,995)

CCI: coeficiente de correlación intraclase; IC: intervalo de confianza; todos los CCI mostraron valores de p<0,001.

**Figura 1:**
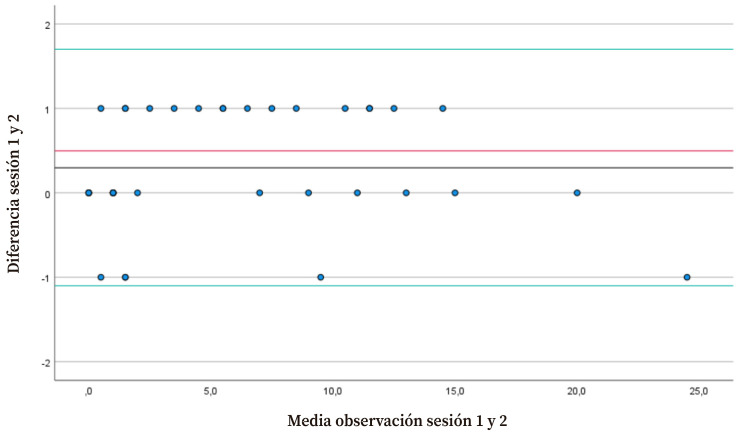
Gráfico de Bland & Altman para la fiabilidad intraobservador del total de la escala. La línea roja es la media de la diferencia entre las dos puntuaciones del mismo observador; las líneas verdes son los limites de tolerancia superior (1,70) e inferior (-1,10).

Todas las observaciones se encontraban dentro de los límites de tolerancia según el gráfico de Bland y Altman (Fig. 1). La media de la diferencia de las observaciones realizadas entre las sesiones 1 y 2 fue de 0,29 (DE=0,70). Por tanto, los límites de tolerancia de la fiabilidad intraobservador para el total de la escala fueron 1,70 (superior) y -1,10 (inferior), con una amplitud de ±1,4 El SEM y el CMD_95_ de la puntuación total de la escala entre las dos sesiones fueron de 0,044 y 0,12 puntos, respectivamente. 

**Figura 2:**
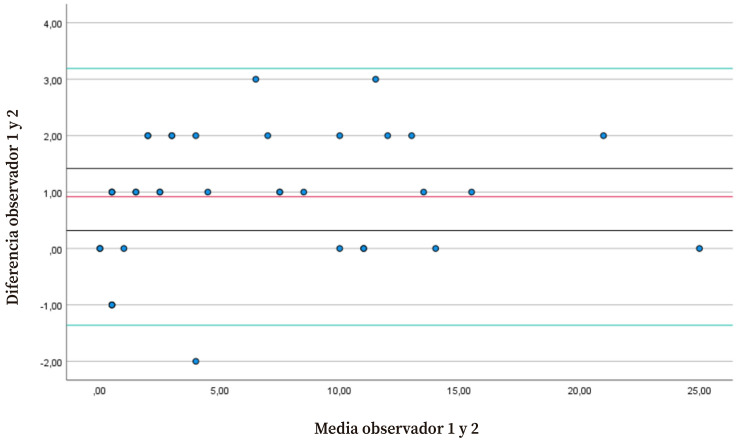
Gráfico de Bland & Altman para la fiabilidad interobservador en dos sesiones del total de la escala. La línea roja es la media de la diferencia entre las puntuaciones de los observadores 1 y 2; las líneas verdes son los limites de tolerancia superior (3,19) e inferior (-1,36).

De acuerdo al gráfico de Bland y Altman, la mayoría de las observaciones realizadas por los dos evaluadores se encontraban dentro de los límites de tolerancia (Fig. 2). La media de la diferencia de las puntuaciones realizadas por los observadores 1 y 2 -que establece el error- fue de 0,918 (DE=1,13), con unos límites de tolerancia de la fiabilidad interobservador para el total de la escala de 3,19 (superior) y -1,36 (inferior), con una amplitud de ±2,28. El SEM y el CMD_95_ de la puntuación total de la escala entre los dos observadores fue de 0,13 y 0,36 puntos, respectivamente. 

### Análisis de la validez de constructo

La puntuación media en la escala de Houghton fue 10,27 (DE=1,55), y mostró una correlación negativa fuerte y estadísticamente significativa (r = -0,773; IC95%: -0,877 a -0,598; p < 0,001) con la escala OMSAEI. Esta correlación inversa es clínicamente coherente: mayores puntuaciones en la escala de Houghton se asocian a mayor funcionalidad e independencia en el uso de la prótesis, mientras que mayor puntuación en la escala observacional indica mayor presencia de alteraciones en el patrón de marcha. 

## DISCUSIÓN

Nuestros resultados muestran una excelente fiabilidad inter e intraobservador de la escala OMSAEI. Además, evidencian una correlación fuerte con la escala de Houghton, que permite evaluar el uso de la prótesis en personas con AMI. 

La escala OMSAEI ha demostrado ser capaz de reproducir de forma consistente las desviaciones cinemáticas y espaciotemporales de la marcha de sujetos con AMI en diferentes sesiones y entre distintos observadores, registrando hallazgos que tradicionalmente se valoraban de forma cualitativa y no estructurada. Este avance resulta especialmente relevante en contextos de rehabilitación de personas con amputación, donde la toma de decisiones clínicas requiere herramientas válidas y reproducibles que aporten criterios comparables entre evaluadores y sesiones. Estudios similares, realizados en personas con amputación, adolecen de una muestra significativa^13^.

Los altos valores de CCI entre todas las puntuaciones indican un excelente acuerdo tanto entre evaluadores como en mediciones repetidas por el mismo profesional. No obstante, el CCI ha sido criticado por ser un valor adimensional, lo que dificulta su interpretación. Los gráficos de Bland-Altman pueden ser más útiles que el CCI, ya que pueden interpretarse de forma clara y significativa tanto en entornos de investigación como clínicos^22^^,^^23^. La amplitud de los límites de acuerdo resulta especialmente útil para evaluar el grado de concordancia o discrepancia entre observadores, mediciones o sistemas, proporcionando una estimación más precisa que el CMD_95_ en algunos contextos^17^. Los gráficos de Bland-Altman mostraron discrepancias de pequeña magnitud tanto para la fiabilidad intraobservador como interobservador. Estos valores pueden considerarse como una referencia para interpretar el sesgo entre evaluaciones realizadas con la escala OMSAEI y podrían entenderse de forma análoga al CMD. De forma coherente con ello, los CMD obtenidos para la escala OMSAEI reflejaron igualmente una variabilidad reducida tanto en la fiabilidad intraobservador como en la interobservador.

En comparación con otras escalas de análisis visual diseñadas para las personas con amputación, como la POGS, la OMSAEI presenta la ventaja de disponer de una excelente validez de contenido^7^ y de incluir ítems que tratan de responder a las alteraciones biomecánicas específicas de la marcha de las personas con AMI que atienden a los parámetros espaciotemporales y cinemáticos. Los pacientes con AMI suelen adoptar movimientos compensatorios durante la marcha, involucrando segmentos como el tronco, la pelvis y la cadera. Entre estos movimientos se incluyen, la circunducción de la cadera y la elevación de la pelvis durante la fase de oscilación para facilitar el avance de la prótesis, la inclinación lateral del tronco hacia el lado protésico para aumentar la sensación de estabilidad, y una mayor basculación pélvica, en el plano sagital, con el fin de mantener la longitud de zancada y mejorar el equilibrio^5^^,^^9^^,^^24^^,^^25^. Además, se han identificado alteraciones en los parámetros espaciotemporales, como disminución de la velocidad de marcha y aumento en la anchura del paso^9^. Todas estas modificaciones podrían ser evaluadas mediante la escala OMSAEI.

Como ha sido descrito, la asimetría del patrón de marcha en la persona con AMI repercute en su calidad de vida y es responsable de generar complicaciones osteoarticulares. Por ello, la evaluación de estas personas a lo largo del tiempo debería incluir escalas validadas en población con AMI^26^^-^^29^, que ofrezcan una visión específica del paciente y de su evolución que permita diseñar estrategias de intervención adecuadas, como la escala OMSAEI, siendo necesarias investigaciones que evalúen la validez de constructo de esta y otras escalas. 

Existen otras herramientas de análisis observacional de la marcha. La POGS constituye la primera escala desarrollada específicamente para población con amputación, con 16 ítems dirigidos a caracterizar desviaciones protésicas. Su estudio de repetibilidad en amputados unilaterales mostró una fiabilidad intraobservador superior a la interobservador, especialmente cuando se empleaba registro en vídeo. Sin embargo, su fiabilidad interobservador era variable (de pobre a moderada según los parámetros analizados) y aún carece de estimaciones claras de CMD, además de haberse evaluado en muestras pequeñas^12^^,^^30^. La GAIT dispone de un cuerpo de evidencia amplio en poblaciones neurológicas, mostrando excelente fiabilidad intra e interobservador, y adecuada sensibilidad al cambio tanto en pacientes con ictus como con esclerosis múltiple^13^^,^^17^, pero no ha sido estudiada específicamente en personas con amputación. Por tanto, la OMSAEI se sitúa como una alternativa específicamente concebida para la población con amputación y con propiedades métricas robustas, complementando las limitaciones observadas en POGS y la falta de validación de GAIT en personas con amputación.

Este estudio presenta varias limitaciones. Primero, el diseño observacional transversal, realizado en un único momento temporal impide evaluar la sensibilidad al cambio de la escala y su capacidad para detectar progresos o retrocesos tras intervenciones terapéuticas. Segundo, la muestra se obtuvo mediante un muestreo no probabilístico en un único centro, lo que restringe la generalización de los hallazgos. Tercero, aunque el tamaño muestral supera el mínimo recomendado para estudios de fiabilidad, su distribución heterogénea, con AMI unilateral, principalmente transfemorales y transtibiales, limita su validez en otros niveles de amputación o en amputaciones bilaterales. Además, no se realizó una prueba de validez de criterio frente a un análisis 3D del movimiento, lo que constituye una limitación importante en la evaluación de la escala.

Por ello, futuros estudios deben incluir en su análisis un estudio de fiabilidad y validez de las puntuaciones obtenidas de la observación del lado intacto o contralateral al lado protetizado, un análisis más profundo de la validez de constructo, un análisis de la validez de criterio (respecto a prueba de referencia de análisis 3D del movimiento) y un estudio de sensibilidad, para así completar las propiedades psicométricas de la escala OMSAEI. De igual modo, resulta pertinente desarrollar líneas de investigación más definidas, entre ellas la validación transcultural (al inglés u otros idiomas) y estudios longitudinales destinados a evaluar su estabilidad temporal.

Desde una perspectiva clínica, la OMSAEI ofrece un método estructurado para identificar limitaciones funcionales y orientar la planificación individualizada del tratamiento. Su fiabilidad respalda su uso en el seguimiento evolutivo, permitiendo detectar cambios relevantes y ajustar las intervenciones de forma precisa. Además, su formato estandarizado facilita la comunicación entre profesionales y su aplicación sencilla la hace adecuada para entornos asistenciales con altas demandas de tiempo.

En conclusión, la escala OMSAEI ha demostrado una excelente fiabilidad inter e intraobservador para la evaluación del lado protetizado, lo que respalda su consistencia en la evaluación de patrones de marcha en personas con amputación unilateral. Además, mostró una fuerte correlación negativa con la escala de Houghton, lo que muestra su validez convergente como herramienta complementaria para valorar el uso funcional de la prótesis. Los resultados obtenidos mediante los gráficos de Bland-Altman y los valores de CMD_95_ indican que el error de la escala es pequeño. Estos hallazgos del presente trabajo respaldan la aplicabilidad clínica de la escala OMSAEI y avalan su recomendación como herramienta sistemática para la evaluación observacional de la marcha en personas con AMI.

## Data Availability

Se encuentran disponibles bajo petición al autor de correspondencia.
